# Self-Management Characterization for Families of Children With Medical Complexity and Their Social Networks: Protocol for a Qualitative Assessment

**DOI:** 10.2196/14810

**Published:** 2020-01-23

**Authors:** Rupa S Valdez, Christopher Lunsford, Jiwoon Bae, Lisa C Letzkus, Jessica Keim-Malpass

**Affiliations:** 1 Department of Public Health Sciences School of Medicine University of Virginia Charlottesville, VA United States; 2 Department of Orthopaedics School of Medicine Duke University Durham, NC United States; 3 School of Nursing University of Virginia Charlottesville, VA United States

**Keywords:** children with medical complexity, care coordination, social network, qualitative description, health care self-management, family management, multiadic analysis, contextual environment

## Abstract

**Background:**

Children with medical complexity (CMC) present rewarding but complex challenges for the health care system. Transforming high-quality care practices for this population requires multiple stakeholders and development of innovative models of care. Importantly, care coordination requires significant self-management by families in home- and community-based settings. Self-management often requires that families of CMC rely on vast and diverse social networks, encompassing both online and offline social relationships with individuals and groups. The result is a support network surrounding the family to help accomplish self-management of medical tasks and care coordination.

**Objective:**

The goal of this study is to use a theoretically driven perspective to systematically elucidate the range of self-management experiences across families of CMC embedded in diverse social networks and contextual environments. This approach will allow for characterization of the structure and process of self-management of CMC with respect to social networks, both in person and digitally. This research proposal aims to address the significant gaps in the self-management literature surrounding CMC, including the following: (1) how self-management responsibilities are distributed and negotiated among the social network and (2) how individual-, family-, and system-level factors influence self-management approaches for CMC from a theoretically driven perspective.

**Methods:**

This study will encompass a qualitative descriptive approach to understand self-management practices among CMC and their social networks. Data collection and analysis will be guided by a theoretical and methodological framework, which synthesizes perspectives from nursing, human factors engineering, public health, and family counseling. Data collection will consist of semistructured interviews with children, parents, and social network members, inclusive of individuals such as friends, neighbors, and community members, as well as online communities and individuals. Data analysis will consist of a combination of inductive and deductive methods of qualitative content analysis, which will be analyzed at both individual and multiadic levels, where interview data from two or more individuals, focused on the same experience, will be comparatively analyzed.

**Results:**

This study will take approximately 18 months to complete. Our long-term goals are to translate the qualitative analysis into (1) health IT design guidance for innovative approaches to self-management and (2) direct policy guidance for families of CMC enrolled in Medicaid and private insurance.

**Conclusions:**

Multiple innovative components of this study will enable us to gain a comprehensive and nuanced understanding of the lived experience of self-management of CMC. In particular, by synthesizing and applying theoretical and methodological approaches from multiple disciplines, we plan to create novel informatics and policy solutions to support their care within home and community settings.

**International Registered Report Identifier (IRRID):**

PRR1-10.2196/14810

## Introduction

Children with medical complexity (CMC) are a growing population of medically fragile children with complex, multisystem disease states, technology dependence, severe functional limitations, complicated treatment regimens and therapies, and surgical interventions that require comprehensive self-management [1-3]. Advancements in neonatal and pediatric critical care, nutrition therapies, and emerging technologies have resulted in improved survival rates among medically fragile children [2]. These children are often left with life-threatening complex systemic health problems, including neurodevelopmental disabilities, gastrointestinal limitations, pulmonary and cardiac complications, and musculoskeletal abnormalities, among other issues [2]. CMC often require 24-hour-a-day monitoring and care, frequently occurring in home- and community-based settings and relying on immense caregiving efforts [4].

In the context of CMC, self-management can be conceptualized as family management, or care where the family —along with the child in some cases—is actively participating in the child’s health management. Such self-management requires coordinating and implementing the instructions of numerous providers, which may include medications, therapies, home monitoring, technologic dependence, and complex care plans [5]. Beyond medical aspects, families of CMC must also engage in emotional management and role navigation management [6,7]. This range of activities occurs within variously configured contextual environments (eg, technological, community, and physical) that contribute to or act as barriers to high-quality self-management [8]. For example, patient portals from electronic health records and other digital tools present ample opportunities for improved care coordination. Yet, to date, implementation of such tools has not been robust, perhaps due to practice variations, regional restrictions, and reimbursement obstacles [9]. The current fragmented nature of care coordination and family-centered navigation suggests that the development of consumer health IT to enable these practices is in early stages, and the various technologies that do exist for this purpose are often not aligned with the realities of families’ lives [10].

Despite CMC encompassing several diagnoses, the self-management needs and practices among all CMC and their families are shared, so it is critically important to contextualize CMC as a whole [11]. Moreover, to meet self-management challenges, families of CMC likely rely on vast and diverse social networks [11], defined as the web of social relationships surrounding an individual [12]. Involving social network members in self-management requires ongoing engagement and communication or sharing content through various media with multiple individuals [10]. Since these social relationships can exist either offline or online, there may be additional opportunities for innovative digital care coordination interventions.

**Figure 1:**
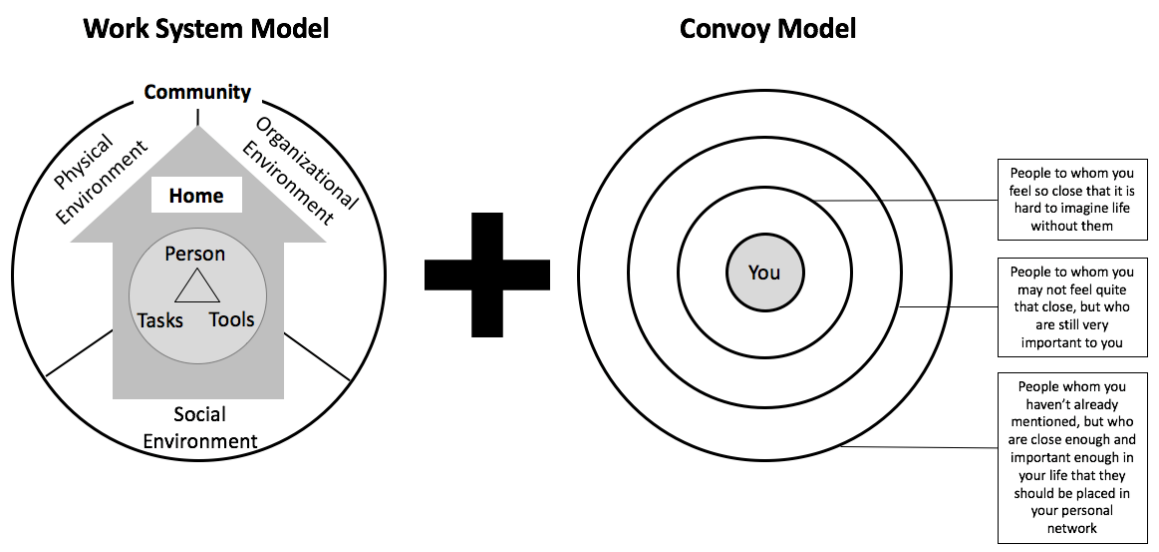
Theoretical underpinnings of the study involving the work system model and convoy model.

The theoretical framework guiding this study is synthesized from literature in human factors engineering, nursing, and public health. The foundation of the theoretical framework is drawn from the public health structure-process-outcome model proposed by Donabedian [13]. It asserts that the home and community settings in which self-management of CMC occurs (ie, structure) impact the ways in which self-management is enacted (ie, process). These self-management processes, in turn, affect the health status of CMC (ie, outcome) [13-15]. The structure of home and community settings is grounded in work system theory, drawn from human factors engineering, which specifies that such settings are characterized by individual (ie, patient and others involved in his or her care) attributes (eg, literacy and age), tasks (eg, complexity and frequency), tools and technology (eg, access and usability), physical environment (eg, clutter and noise), social environment (eg, social support), and organizational environment (eg, routines and policies) [16-19]. The theoretical framework further emphasizes the primacy of the social environment, explicitly accounting for the structure of the affective social networks and the roles played by each social network member (see Figure 1). We draw on the convoy model from the nursing literature to explicate the structure of the social environment [10]. This model has been adopted and adapted for use in other similar studies seeking to understand the role of the social environment in health [10].

Self-management processes shaped by these structural components, and drawn from the nursing literature, include learning about the condition and health needs, recognizing and managing body responses, identifying and benefiting from psychological resources, processing and sharing emotions, and obtaining and managing social support, among others. Proximal and distal outcomes impacted by these self-management processes include those that are related to health status, quality of life, relationship dynamics, and cost of care [20].

This study focuses on structure and process, while follow-up studies will be extended to focus on outcomes. We view this study as a critical step in understanding family and social engagement surrounding self-management of patients with medical complexity. As consumer health information technologies are created to support family engagement in the self-management process, there is a critical need to ensure that the technologies, which are intended to support families, function in ways that families are working and communicating [10]. Thus, we will first focus on connecting all aspects of the work system to the experiences of self-management processes, while we will subsequently focus on more deeply connecting the social environmental component of the work system to self-management processes through a social network lens.

In summary, this research proposal aims to address the significant gaps in the self-management literature surrounding CMC, including the following: (1) how self-management responsibilities are distributed and negotiated among the social network and (2) how individual-, family-, and system-level factors influence self-management approaches for CMC from a newly synthesized theoretical perspective. This view includes individual and family factors within the context of people’s social, technical, and environmental lives, while also systematically exploring perspectives from members of the CMC’s network.

## Methods

### Overview

This study takes a qualitative descriptive approach [21,22] to understand self-management practices among CMC. The qualitative descriptive approach focuses on generating a comprehensive summary of events with presentation of interpretive categories in everyday language [22]. Given that qualitative descriptive studies may begin with an overarching framework [21], data collection and analysis will be guided by the combined theoretical framework described above. However, both data collection and analysis will remain open to the data; in other words, it is possible that the concepts of interest will change over time [21,22]. Data collection will consist of semistructured interviews with children, parents, and social network members. Data analysis will consist of a combination of inductive and deductive methods of qualitative thematic analysis [23], which will use data generated by interviews and application of line-by-line codes; relevant categories to generate final themes will then be used describe the phenomena of interest. Relevant categories and themes will be analyzed at both individual and multiadic levels (ie, intragroup) [21,22] to understand similarities and differences within each case (ie, child) in terms of the various facets of the family and social networks and across cases regarding the same. Further, multiadic analysis means that relevant comparisons and contrasts will be made across similar thematic domains within each case. We expect there to be a diversity in disease representations but a shared experience in self-management burdens and difficulty in navigation between a multitude of settings.

Eligible families will initially be approached to participate in this study from an academic children’s hospital that provides specialist inpatient and outpatient services, including a pediatric complex care clinic. Each family will be comprised of a child between 0 and 21 years of age with diagnoses and/or a clinical presentation consistent with the definition of medical complexity, as well as the child’s parents. Operationalization of CMC will follow the Center of Excellence on Quality of Care Measures for Children with Complex Needs (COE4CCN) definition of the highest level of medical complexity of children with complex chronic disease [24]. This definition includes any of the following scenarios: significant chronic conditions in two or more body systems where the physical, mental, or developmental conditions have lasted at least a year; use of health care resources are above the level for a healthy child; and treatment is required for control of the condition. In addition, the conditions are expected to meet one of the three following descriptions: episodic or continuously debilitating, a progressive condition that is associated with deteriorating health with a decreased life expectancy in adulthood, child requires continuous dependence on technology for at least 6 months, or a progressive or metastatic malignancy that impacts life function. Maximum variance sampling [25] will be used to recruit participants; this will be based on condition as well as demographic characteristics shown to impact child self-management, including parent and child gender, age, race or ethnicity, geographic location, health literacy, educational background, and health status. This sampling strategy will enable us to gain insight into a wide range of established approaches to self-management in children with medical complexity.

If a child is unable to provide assent or consent, only the parents will be included. Furthermore, if only one parent is present or willing to participate, only this individual will be included. Thus, a family is eligible to participate in this study in any of the following combinations of individuals: a child and two parents, a child and one parent, two parents, or one parent. In this study, we refer to a social network set as the individual social networks of each family member. Thus, a social network set could be comprised of only one social network (ie, one parent’s network), two social networks (ie, both parents’ networks or one parental and one child network), or three social networks (ie, both parental networks and one child network). Together, a family and their social network set comprise a case. Social network members will be selected by participants based on prompts associated with the convoy model instrument [10] and may include individuals such as neighbors, friends, members of the community, and members of online social networks, in addition to family members [10]. During the consent process, it will be explained that the investigators are interested in contacting members of the participants’ social network for potential inclusion in the self-management study. Following completion of the parent interviews, participants will be asked for contact information for potential social network recruitment.

### Measures

Semistructured interviews (see Table 1) will be conducted and will consist of four overarching domains. Part 1 of the interview will involve opening questions where both parents and children will answer open-ended questions about their daily routines. Part 2 of the interview process will elicit questions surrounding how self-management responsibilities are distributed and negotiated among the social network. This section of the interview will provide an exploration of participants’ social networks and the ways in which the network is engaged in self-management processes. Participants will be guided through a structured process of explicating their affective social networks [26]. They will be asked a series of open-ended questions to understand the characteristics of each social network member and their general relationship with the social network member. The interview will then transition to self-management. Participants will be asked about their own roles in specific self-management processes and the role of each social network member. Self-management processes include health-related domains of the following: therapeutics (ie, medications, technology dependence, and therapies), care coordination (ie, appointments, information exchange, logistics like transportation, and home activities of daily living), and management of unexpected health events (ie, side effects, unexpected illnesses, and unexpected hospitalizations).

Once each social network member has been discussed, participants will be asked if there is anyone else who helps with their own or their child’s self-management whom they did not place in their social network. Participants will be asked whether or not these individuals should be placed in the social network and asked questions about each person similar to those above. Finally, participants will be asked to reflect on their network as a whole and the ways in which they engaged this network in specific self-management processes.

Part 3 of the interview will provide an exploration of participants’ work systems and the ways in which work system elements shape self-management. Participants will be asked to think of times in the past when they were engaged in specific self-management processes. Participants will then be asked to reflect on how different aspects of their work system contributed to these experiences; these aspects include the following: personal attributes (eg, literacy, health literacy, education, socioeconomic status, and age), tasks (eg, complexity and frequency), technology (eg, access to and usability of personal health records, automated reminders, and health-related apps), physical environment (eg, clutter and noise), social environment (eg, support from social networks inclusive of individuals such as family members, friends, home care aides, and health care providers), community environment (eg, public transportation), and health policy environment (eg, insurance, characteristics of the health care delivery system such as financial assistance, and care coordination practices) [16]. A similar structure will be used to understand the challenges that participants have faced when engaging in the same self-management processes.

**Table 1:** Interview domains and sample questions for self-management of children with medical complexity (CMC).

Domain	Details	Sample questions for interviews with CMC and their parents or legal guardians
General		What does a typical day look like? What makes some days easier than others?
Social network member characteristics	Identification, characteristics, and stability	Can you tell me about your relationship with [social network member]? How has your relationship with [social network member] changed over time? What do you think makes [social network member] a part of your social network?
Self-management	Domains include therapeutics, care coordination, and management of unexpected health event Aim 1: How self-management responsibilities are distributed and negotiated among the social network	Now we are going to talk about taking care of your/your child’s medication needs. This includes everything from getting a prescription from your provider, filling the prescription, and taking the medication as directed. What do you do to take care of your/your child’s medication needs? Please tell me about a time when you were taking care of your/your child’s medication needs/treatment and felt things went well? Please tell me about a time when you were taking care of your/your child’s medication needs and felt things went poorly?
Social network member self-management involvement	Corresponding with self-management domains Aim 1: How self-management responsibilities are distributed and negotiated among the social network	How does [social network member] help take care of your/your child’s medication needs? Why are they involved in that way? How do you feel about their involvement?
Reflection on social network as a whole	Aim 1: How self-management responsibilities are distributed and negotiated among the social network	How do you decide who to ask for help about [your child’s] management? Who are the first people you reach out to? Why do you reach out to them first?
Work system	Demographics, social determinants, modifications, and financial toxicity Aim 2: How individual-, family-, and system-level factors influence self-management approaches for CMC from a theoretically driven perspective	Now we want to know about how things in your environment (house and community) impact the care of your child. Have there been any modifications to the home that help you/your child accomplish activities of daily living? What kind of access to you have to the Internet? Have you moved around a lot from your current home?

Interviews with social network members will also consist of four parts. In addition to opening and concluding questions that mirror those above and include demographic questions, the following questions related to the two aims will be asked. Questions will be oriented to the individuals that placed the social network member in their network (eg, child only or both parents and child). Social network members will be asked general questions about their relationship with the child and/or parents. They will also be asked where in their social network they would place the parent or child. Social network members will then be asked about their role in managing the child’s health, how they assumed that role, and how they feel about that role. Social network members will be asked about times in the past when they were involved in managing specific aspects of the child’s health and felt that things went well. They will then be asked to reflect on how different aspects of the parent or child’s work system (as described in Table 1) contributed to these experiences. Similar questions will be asked for when things were not perceived to go well. Follow-up questions will be skipped if a social network member does not engage in a specific aspect of managing the child’s health.

### Outcomes and Data Analysis

Analyses for both aims will begin with cross-sectional analysis, will move to within-case comparisons, and will end with across-case comparisons. These analyses will build on one another. That is, the cross-sectional analysis will be used to generate prevalent analytical categories and themes within the data. Within-case analysis will then be used to determine how these themes manifest and interact within each case. Finally, across-case analysis will be used as a higher order cross-sectional analysis that will allow for a meta-analysis of how these categories and themes manifest and interact across families and social networks.

Two types of cross-sectional views will be created: one drawn from all parent and child data and one drawn from all social network member data. In addition, analyses will be conducted both by self-management process and in aggregate across all self-management processes. Regarding the *parent and child view*, we will begin by describing parent and child social network structure and composition. Descriptive statistics (eg, demographics of social network members) and conventional qualitative content analysis [23], in which themes are derived from data (eg, relationship with social network members), will be used to develop cross-sectional views of these social networks. This latter method will also be used to analyze open-ended questions about the nature of self-management responsibilities and how they are distributed and negotiated among social network members. We will then use directed qualitative thematic analysis [23], in which operational definitions for initial categories and subcategories are determined using theory. The work system model and relevant empirical studies will be used to develop initial categories of barriers and facilitators. Regarding the *social network view*, analysis will parallel that described for the *parent and child view*, resulting in an aggregate view and views by self-management process. As described above, conventional qualitative content analysis will be used to analyze open-ended questions.

Methods of multiadic analysis will be used to create two types of within-case comparisons [27,28]. The first type of within-case comparison will occur at the level of the family. When two or three individuals participate from one family (ie, both parents, both parents and child, or one parent and child), multiadic methods will be used to compare the responses provided by each individual. In particular, points of overlap and difference between viewpoints will be explicitly coded using a combination of directed and conventional qualitative content analysis (ie, deductive and inductive methods). Drawing on Eisikovits and Koren’s framework [28], we will first code into four themes: (1) overlap on open and hidden reality, (2) contrast on open and hidden reality, (3) contrast on open reality and overlap on hidden reality, and (4) overlap on open reality and contrast on hidden reality. Open reality refers to what is described by individuals (eg, the event that occurred), whereas hidden reality refers to the ascribed meaning (eg, how the event was interpreted). This four-dimensional framework will be extended in the case of the participation of three family members. Within this framework, categories drawn from the data will be created to account for types of open and hidden realities. The second type of within-case comparison will occur at the level of the social network. A dyadic analysis will take place comparing the narratives of the family member with the narratives of each of their social network members (eg, What does the mother say about her own role and her sister’s role, and what does her sister say about her own role and the mother’s role?). This analysis will occur as described above. Another iteration of analysis will then be conducted to reduce the dyadic analyses into a synthesized view for each family member. For example, for each of 10 individuals in a child’s social network, a dyadic analysis will be conducted that will result in material under each of the four themes. The next round of analysis would consist of a qualitative content analysis of all of the material within each theme. Thus, the final analysis for each social network set would consist of an aggregate view of the four themes in Eisikovits and Koren’s framework across all social network members. One aggregate analysis will be created for each family member.

A final structural layer of analysis will be added with across-case comparisons. To create comparable analytic instruments that further reduce the data, we will create individual case reports [29,30]. These reports will be narrative in nature and will highlight key characteristics of each case (eg, number of family members involved, family demographics, and key themes from cross-sectional, dyadic, and multiadic analyses). Case reports will be comparatively analyzed using conventional qualitative content analyses. Guiding questions relevant to this analysis will include the following: (1) Which approaches to distributing and negotiating self-management responsibilities are common and distinct across families and their social networks? and (2) How do families and social networks with apparently similar characteristics differentially engage in distributing and negotiating self-management practices? Analyses guided by these questions will enable a nuanced understanding of mechanisms driving specific self-management experiences within and across various social networks. For all analysis steps, all investigators will individually analyze 20% of the data before reaching a consensus. Consensus guidelines will be documented for use in subsequent analyses. The remaining analyses will be divided between the principal investigators, who have in-depth experience with both forms of qualitative content analysis to be used in the study. Analysis will be discussed at regular full-team meetings. All analyses will be conducted using NVivo 11 (QSR International). Reflective journal entries will be maintained by the lead investigators during the research process. Methods to ensure rigor and trustworthiness [31] of the data analysis will include aspects of demonstrating (1) credibility (ie, direct observation of online communication, iterative questioning, and frequent debriefing), (2) transferability (ie, contextual review), (3) dependability (ie, maintaining an audit trail), and (4) confirmability (ie, bracketing, investigator triangulation, and member checking through the stakeholder advisory board). The key decision points for the qualitative research analysis will also be assessed and recorded in an online platform through the Center for Open Science to ensure rigor and reproducibility. Anticipated limitations of this study design include the following: lack of generalizability based on recruitment from a single-site study and potential for recall bias with self-reported data. Additionally, we lack the ability to triangulate self-management experiences within the school or other immersive environments.

## Results

We anticipate that this project will take nearly 18 months, including recruitment, interviewing, and completion of data analysis. Challenges to the protocol may involve study recruitment challenges; feasibility of the completion of long parent interviews, while caring for CMC; and analytic integration of multiadic components. To offset these potential challenges, we will consider opening the recruitment to online methods, offering participants the ability to complete the interviews in more than one session, and developing network mapping approaches to be applied to data analysis to offset the sheer quantity of data and analytics lenses. Findings from this study will be disseminated to academics, clinicians, and policy makers. Our long-term goal is to translate the qualitative analysis into (1) health IT design guidance for innovative approaches to self-management and (2) direct policy guidance for families of CMC enrolled in Medicaid and private insurance.

## Discussion

This study is theoretically, conceptually, and methodologically innovative. First, the theoretical framework is innovative in nature, in that it synthesizes nursing, human factors engineering, and public health perspectives. In doing so, it elaborates upon existing theoretical frameworks used to guide the study of self-management. Our model is grounded in Donabedian’s structure-process-outcome assessment model [13-15]. Key concepts and relationships derived from this public health sciences model are present in general models of self-management [32,33] and those that are specific to children with chronic conditions [34,35], demonstrating the relevance of Donabedian’s model for conceptualizing self-management. Previous models, however, are not explicitly grounded in additional theoretical perspectives. We address these limitations by integrating nursing and human factors engineering theories into Donabedian’s model. Integration of the convoy model [26] from nursing enables explicit consideration of the social network. Integration of a synthesized work system model from human factors engineering [16] enables explicit consideration of all structural elements in previous self-management models, in addition to elements empirically shown to impact self-management [8] but not included in these previous models. This integration of the work system model with another model is consistent with a novel approach within human factors engineering [36].

Second, this study is innovative in extending the conceptualization of the “who” in self-management. A traditional definition of self-management focuses on an individual’s solitary actions or actions that require interacting with members of the formal health care delivery system [37]. More recently, the definition of self-management has been extended by many, including the National Institutes of Health (NIH) [38], to encompass the actions of the individual’s family members. This study augments this conceptualization in two ways. First, it explicitly accounts for the extended social network of the individual with a chronic condition, enabling systematic understanding of the ways in which individuals beyond family members are engaged in health management. Such an approach will allow us to understand the potentially limited but essential roles played by a broader range of actors. Second, this study explicitly includes the extended social network not only of the individual with a chronic condition, but also of the primary caregivers. This expanded approach will foster a more holistic understanding of how self-management relies not only on a patient’s social network, but also on the social networks of the primary informal caregivers.

Methodologically, this study is innovative in adopting an analytic approach from counseling psychology and family communication. To our knowledge, such an approach has not been applied to studies of self-management nor of CMC. Namely, this study leverages methods of dyadic and multiadic analysis—in which interview data from two or more individuals, focused on the same experience, is comparatively analyzed—to explicitly account for multiple perspectives on CMC self-management. These methods of qualitative analysis [27] have the potential to reveal points of agreement, disagreement, and differential understanding among individuals [28]. Points of misunderstanding and disagreement may then be conceptualized as points of intervention. It is important to underscore that such an approach is distinct from approaches that comparatively analyze all patient data with all family caregiver data. Such an approach compares narratives only at the aggregate level. The approach of this study is comparative, both at the dyad level as well as at the aggregate level. In conclusion, multiple innovative components of this study will enable us to gain a comprehensive and nuanced understanding of the lived experience of self-management of CMC and an understanding of the spillover impacts experienced by families of CMC and members of their social networks. In particular, by synthesizing and applying theoretical and methodological approaches from multiple disciplines, we plan to create novel informatics and policy solutions to support care within home and community settings. Subsequent studies will (1) enable us to further investigate how diverse lived experiences are tied to health outcomes and (2) enable us to determine how innovative health IT and policy solutions may alter these lived experiences to positively affect health outcomes.

## Appendix

Multimedia Appendix 1 Peer review reports from the University of Virginia.

## Abbreviations

CMC: children with medical complexity
COE4CCN: Center of Excellence on Quality of Care Measures for Children with Complex Needs
NIH: National Institutes of Health
NINR: National Institute of Nursing Research
