# Supplementary material for: Self-Management Characterization for Families of Children With Medical Complexity and Their Social Networks: Protocol for a Qualitative Assessment
Source: JMIR Res Protoc. 2020 Jan 23;9(1):e14810. doi: 10.2196/14810 (PMC7005691; doi:10.2196/14810)
Supplement: Multimedia Appendix 1 [file resprot_v9i1e14810_app1.pdf]

SUMMARY STATEMENT
(Privileged Communication)

PROGRAM CONTACT:
Karen Huss
301.594.5970
hussk@mail.nih.gov

Release Date: 06/18/2018
Revised Date:

Application Number: 1 R21 NR017991-01A1

Principal Investigators (Listed Alphabetically):

KEIM -MALPASS, JESSICA KEIM (Contact)
VALDEZ, RUPA SHETH

Applicant Organization: UNIVERSITY OF VIRGINIA

Review Group: ZRG1 NRCS-V (08)
Center for Scientific Review Special Emphasis Panel
Nursing and Related Clinical Sciences

Meeting Date: 05/31/2018
Council: OCT 2018
Requested Start: 09/01/2018
RFA/PA: PA18-384
PCC: ACCKH

Project Title: Self-Management Characterization for Children with Medical Complexity and their Social Networks
SRG Action: Impact Score:16
Next Steps: Visit https://grants.nih.gov/grants/next_steps.htm
Human Subjects: 30-Human subjects involved - Certified, no SRG concerns
Animal Subjects: 10-No live vertebrate animals involved for competing appl.
Gender: 1A-Both genders, scientifically acceptable
Minority: 1A-Minorities and non-minorities, scientifically acceptable
Children: 1A-Both Children and Adults, scientifically acceptable

Project Year	Direct Costs Requested	Estimated Total Cost
1	150,000	242,250
2	125,000	201,875
TOTAL	275,000	444,125

ADMINISTRATIVE BUDGET NOTE: The budget shown is the requested budget and has not been adjusted to reflect any recommendations made by reviewers. If an award is planned, the costs will be calculated by Institute grants management staff based on the recommendations outlined below in the COMMITTEE BUDGET RECOMMENDATIONS section.

1R21NR017991-01A1 Keim-Malpass, Jessica

RESUME AND SUMMARY OF DISCUSSION: RESUME AND SUMMARY OF DISCUSSION: The application proposes to systematically elucidate the range of self-management experiences across families of children with medical complexity (CMC) embedded in diverse social networks and contextual environments. Using a qualitative descriptive approach to understand self-management practices in CMC, analyses will seek to understand agreement, disagreement, and differential understanding among the multiple individuals involved in self-management of CMC. The application was considered to be highly significant with potential to affect policy and improve services to affected families. The research is important because of increased prevalence, and the premise supports the need to increase understanding of this population. The investigative team is strong and the research focus is novel. The approach is theoretically driven, and the design is rigorous. For example, attention is paid to sex as a biological variable, and the qualitative approach is detailed. The main, but minor, weakness is lack of attention to the online nature of many social networks. Aside from this concern, the application was highly responsive to prior critiques. Overall, it is an outstanding application proposing a study of high scientific impact for families of children with medical complexity.

DESCRIPTION (provided by applicant): Children with medical complexity (CMC) are a growing population of medically fragile children with special health care needs (between the ages of birth to 21) with complex, multisystem disease states. Nearly 1 percent of all children (roughly 320,000 to 560,000) have medical complexity at the highest levels, yet these children account for up to a third of all pediatric health care spending and more than half of all unintended readmissions. Much of the family-centered self-management (hereafter self-management) occurs in the home and community-based setting with immense caregiving demands. The goal of this study is to use a theoretically-driven perspective to systematically elucidate the range of self-management experiences across families of CMC embedded in diverse social networks and contextual environments. Previous pediatric research has not articulated self-management experiences in ways that account for multiple diagnoses, engagement of social network members, or simultaneous consideration of individual and environmental factors. In other words, self-management has not been studied through assessment of both individual/family/social network level factors and system level factors simultaneously. Consequently, the aims of this study are: (1) Examine how self-management responsibilities for CMC are distributed and negotiated among multiple social network members and (2) explicate how individual and systems level factors influence approaches to self-management for CMC. This study takes a qualitative descriptive approach to understand self-management practices in CMC and draws on theoretical perspectives from nursing, systems engineering, and public health. It further draws on novel analysis methods that enable understanding of agreement, disagreement, and differential understanding among the multiple individuals involved in self-management of CMC. Future research efforts will include a larger, multisite study which builds upon the findings by assessing changes in self-management practices over time and capturing multiple perspectives on patient-reported distal and proximal outcomes for CMC. Findings from these combined studies will focus on developing and testing interventions (educational, technological, and policy oriented) to determine how different ways of leveraging and configuring social networks and contextual environments of families of CMC impact patient-reported, clinical, and utilization outcomes.

PUBLIC HEALTH RELEVANCE: The goal of this study is to use a theoretically-driven perspective to systematically elucidate the range of self- management experiences across families of children with medical complexity (CMC) embedded in diverse social networks and contextual environments. The long-term objective of this research is to improve the health and quality of life of CMC and their parents/guardians by developing a foundation for interventions focused on enhanced self-management.

CRITIQUE 1

Significance: 2
Investigator(s): 2
Innovation: 1
Approach: 2
Environment: 2

Overall Impact: This is a resubmission of a qualitative study that explores the self-management strategies of families of children with complex medical conditions in the context of social networks. The investigators make the point that despite the significant personal and financial burden of taking care of these children few studies have examined the process of self-management beyond the individual caretakers. The resubmission is responsive to prior critiques. The scientific premise is sound observing that secondary to advances in medical life saving strategies more and more children with complex medical needs are surviving. Understanding the families' self-management strategies, needs, problem solving and quality of life is an important first step in influencing policy and the provision of needed supportive services. The application has a number of strengths including the investigative team, an innovative theory driven design, and a rigorous and well described approach to analysis. Sex a biological variable is appropriately incorporated into the analysis. Weaknesses are minor and addressable. The overall impact of the results of the study are expected to support both families and policy makers.

1. Significance:

Strengths

- The number of children with medical complexity is growing with advance in medicine
- Prior research has primarily explored family self-management on an individual basis
- The consideration of social networks strengthens and supports the scientific premise

Weaknesses

- This study is exploratory and the importance of social networks is hypothesized (minor weakness)

2. Investigator(s):

Strengths

- The PI of this project has both clinical and research expertise with the population of children with complex medical needs and their families
- Valdez brings unique and important research expertise to the project
- Valdez has successfully managed a number of funded research projects
- Multiple PI plan provided

Weaknesses

- A history of collaboration is not evident (minor weakness)

3. Innovation:

Strengths

- Synthesis of theoretical approaches (nursing, public health, systems engineering will guide the research
- Contextualization of self-management in the broader perspective of networks of care

Weaknesses

- None noted

4. Approach:

Strengths

- Strategies to accomplish study activities are well thought out
- Well designed and theory supported approach to analysis
- Trustworthiness of data discussed and supported
- Recruitment is designed to include a range of disabilities and races among the participants
- De-identified qualitative data will be shared on a publicly accessible qualitative library

Weaknesses

- Consideration that in today's electronic world social networks may also be electronic. This is not discussed in the proposal or how it will be addressed. (minor weakness)
- Although the family is proximal and can engage in face to face interviews It is not clear if this will be a limitation for the social network participants identified by the proband family (minor weakness)
- The enrollment table reflects only the children

5. Environment:

Strengths

- Hospital and clinical services at UVA will provide sufficient space and resources for the recruitment of participants
- Letter of support regarding support of recruitment efforts are provided

Weaknesses

- Significant portion of the environment description is unrelated to the conduct of the study (minor)

Study Timeline:

- Not Applicable (No Clinical Trials)

Protections for Human Subjects:

Acceptable Risks and/or Adequate Protections

- All human subjects concerns are addressed in this minimal risk study

Data and Safety Monitoring Plan (Applicable for Clinical Trials Only):

Inclusion of Women, Minorities and Children:

- Sex/Gender: Distribution justified scientifically
- Race/Ethnicity: Distribution justified scientifically
- For NIH-Defined Phase III trials, Plans for valid design and analysis: Not applicable
- Inclusion/Exclusion of Children under 18: Including ages <18; justified scientifically
- Children included

Resubmission:

This submission is responsive to prior critiques

Budget and Period of Support:

Recommend as Requested

CRITIQUE 2

Significance: 2
Investigator(s): 1
Innovation: 2
Approach: 1
Environment: 1

Overall Impact: This revised application focused on identifying constructs within the caregiver networks among children with chronic conditions. The processes by which these systems operate are not well understood. Understanding their complexities is important to provide a foundation to approach/alter care if there are appropriate targets for intervention that will improve distal outcomes. A systems engineering approach is proposed and is appropriate. The qualitative design is rigorous. The approach is sound and now includes children/networks outside the University of Virginia. Sex as a biological variable is included. The enrollment table does not reflect all persons who will be enrolled. If successful, these data will add to the literature and provide a foundation for intervention work to address the needs of the social networks that care for children with complex medical conditions.

1. Significance:

Strengths

- This project will evaluate self-management strategies in the network of caregivers for children with complex medical conditions – to determine what works and how it works and what doesn't work and why.
- Children with complex medical needs have large networks of caregivers.

Weaknesses

- There is little focus on the distal outcome – the application focuses on process which is important but is only the beginning – a better understanding of how these data will be used to designed interventions/ameliorate what is broken would have strengthened the significance of the application.

2. Investigator(s):

Strengths

- Strong appropriate team – extension of ongoing work – adequate engineering health policy and medical personnel on the team

Weaknesses

- None noted

3. Innovation:

Strengths

- The application of the systems engineering approach and use of multiple theoretical perspectives to guide data collection and analysis is innovate and rich.
- Inclusion of healthcare systems factors is important

Weaknesses

- None noted

4. Approach:

Strengths

- Recruiting from populations outside UVA is important to increase diversity.
- The interviewing process and participant recruitment are well described.

Weaknesses

- The enrollment table reflects only the children

5. Environment:

Strengths

- The University and school of nursing have resources to support this study.

Weaknesses

- None noted

Study Timeline:

Not Applicable (No Clinical Trials)

Protections for Human Subjects:

Acceptable Risks and/or Adequate Protections

- No concerns noted

Data and Safety Monitoring Plan (Applicable for Clinical Trials Only):

Not Applicable (No Clinical Trials)

Inclusion of Women, Minorities and Children:

- Sex/Gender: Distribution justified scientifically
- Race/Ethnicity: Distribution justified scientifically
- For NIH-Defined Phase III trials, Plans for valid design and analysis: Not applicable
- Inclusion/Exclusion of Children under 18: Including ages <18; justified scientifically
- Children included

Budget and Period of Support:

Recommend as Requested

CRITIQUE 3

Significance: 1
Investigator(s): 1
Innovation: 1
Approach: 1
Environment: 1

Overall Impact: The proposed project will examine how self-management strategies for children with medical complexity (CMC) are negotiated across caregivers and members of a social network and how this impacts children's health outcomes. There is a strong scientific premise for the proposed project because CMC individuals account for ~1/3 of all pediatric healthcare expenditures. This is a descriptive, qualitative study with very rigorous design and analytical methods. There is a strong integrated theoretical framework underpinning the study. The MPI team is very strong and they have appropriate clinical and personnel resources to recruit the target sample. The PIs were very responsive to the previous critiques resulting in an exceptionally strong application. The proposed project has the potential to yield critical information and advance the science around self-management interventions for CMC.

1. Significance:

Strengths

- The scientific premise of the proposed study is to examine the self-management experiences of children with medical complexities (CMC), their caregivers, and extended social network which to date, have not been well-studied.
- It is posited that effective, integrated family-centered approaches for self-management that predominantly occur in the home and community could reduce the frequency of acute hospitalization and inpatient care.
- Although only ~1% of children are classified as CMC, they account for over 1/3 of all pediatric health care expenditures.
- The proposed project extends the MPIs' previous research by including members of the caregivers' and child's social network in the process of self-management.
- The study is based on integration of theoretical models from nursing, public health, and human factors engineering research.
- If successful, the study will identify potential targets for intervention that could help families improve their children's care and inform policy.

Weaknesses

- None noted.

2. Investigator(s):

Strengths

- This is a MPI team with complementary expertise in health communication, health policy, communication and systems engineering around self-management practices.
- Dr. Lunsford is a physician with expertise in CMC.

Weaknesses

- None noted.

3. Innovation:

Strengths

- Analytic methods expand on traditional qualitative research strategies through a unique examination of multi-dyadic analyses which reveal areas of agreement, disagreement and differential understanding among individuals in the network.
- The proposed project integrates several theoretical perspectives from different disciplines to examine barriers and facilitators of effective self-management
- Little data exist on effective self-management interventions for CMC
- A focus on the extended social network of the primary caregiver provides a more comprehensive picture of the roles of these individuals and how these roles are negotiated.

Weaknesses

- None noted.

4. Approach:

Strengths

- Scientific rigor was demonstrated
- Sex as biological variable (SABV) was addressed
- Nicely detailed description of the qualitative approach and analytical strategy is provided and will be led by PI Valdez who has expertise in human factors engineering and cultural anthropology
- Recruitment strategy is designed to enhance heterogeneity of participants
- The focus on family management and extended social network who all play an active role in the child’s healthcare management will identify how this network may improve the children’s health outcomes.
- Consideration of caregivers’ barriers (e.g., work, transportation) which impact their ability to optimally manage their child’s healthcare needs

Weaknesses

- The enrollment table(s) should also include the distribution of caregivers and members of the social network.

5. Environment:

Strengths

- The UVA School of Nursing and Department of Public Health Sciences offer acceptable personnel, equipment, and facilities to support the proposed project.
- Strong letters of support from the clinical sites participating for purposes of recruitment.

Weaknesses

- None noted

Study Timeline:

Not Applicable (No Clinical Trials)

Protections for Human Subjects:

Acceptable Risks and/or Adequate Protections

- An appropriate Human Subjects Protection Plan is included

Data and Safety Monitoring Plan (Applicable for Clinical Trials Only):

Not Applicable (No Clinical Trials)

Inclusion of Women, Minorities and Children:

- Sex/Gender: Distribution justified scientifically
- Race/Ethnicity: Distribution justified scientifically
- For NIH-Defined Phase III trials, Plans for valid design and analysis: Not applicable
- Inclusion/Exclusion of Children under 18: Including ages <18; justified scientifically
- Female participants will include children, caregivers and members of the social network
- No racial/ethnic group will be excluded. Purposeful recruitment of racial and ethnic minorities will occur through maximum variance sampling.
- The focus of the proposed project is on children with medical complexity so their inclusion is scientifically justified

Resubmission:

- The PIs were very responsive to the previous critiques resulting in an exceptionally strong application.

Resource Sharing Plans:

Acceptable

- An acceptable data sharing plan is included

Authentication of Key Biological and/or Chemical Resources:

Not Applicable (No Relevant Resources)

Budget and Period of Support:

Recommend as Requested

THE FOLLOWING SECTIONS WERE PREPARED BY THE SCIENTIFIC REVIEW OFFICER TO SUMMARIZE THE OUTCOME OF DISCUSSIONS OF THE REVIEW COMMITTEE, OR REVIEWERS' WRITTEN CRITIQUES, ON THE FOLLOWING ISSUES:

PROTECTION OF HUMAN SUBJECTS: ACCEPTABLE

INCLUSION OF WOMEN PLAN: ACCEPTABLE

INCLUSION OF MINORITIES PLAN: ACCEPTABLE

INCLUSION OF CHILDREN PLAN: ACCEPTABLE

COMMITTEE BUDGET RECOMMENDATIONS: The budget was recommended as requested.

Footnotes for 1 R21 NR017991-01A1; PI Name: Keim -Malpass, Jessica Keim

NIH has modified its policy regarding the receipt of resubmissions (amended applications). See Guide Notice NOT-OD-14-074 at http://grants.nih.gov/grants/guide/notice-files/NOT-OD-14-074.html. The impact/priority score is calculated after discussion of an application by averaging the overall scores (1-9) given by all voting reviewers on the committee and multiplying by 10. The criterion scores are submitted prior to the meeting by the individual reviewers assigned to an application, and are not discussed specifically at the review meeting or calculated into the overall impact score. Some applications also receive a percentile ranking. For details on the review process, see http://grants.nih.gov/grants/peer_review_process.htm#scoring.

MEETING ROSTER

Center for Scientific Review Special Emphasis Panel
CENTER FOR SCIENTIFIC REVIEW
Nursing and Related Clinical Sciences

ZRG1 NRCS-V (08)
05/31/2018 - 06/01/2018

Notice of NIH Policy to All Applicants: Meeting rosters are provided for information purposes only. Applicant investigators and institutional officials must not communicate directly with study section members about an application before or after the review. Failure to observe this policy will create a serious breach of integrity in the peer review process, and may lead to actions outlined in NOT-OD-14-073 at https://grants.nih.gov/grants/guide/notice-files/NOT-OD-14-073.html and NOT-OD-15-106 at https://grants.nih.gov/grants/guide/notice-files/NOT-OD-15-106.html, including removal of the application from immediate review.

CHAIRPERSON(S)

WEISS, SANDRA JEAN, PHD
PROFESSOR AND ESCHBACH ENDOWED CHAIR
COMMUNITY HEALTH SYSTEMS
SCHOOL OF NURSING
UNIVERSITY OF CALIFORNIA, SAN FRANCISCO
SAN FRANCISCO, CA 94143

MEMBERS

APARASU, RAJENDER R, PHD
PROFESSOR AND CHAIR
DEPARTMENT OF PHARMACEUTICAL
HEALTH OUTCOMES AND POLICY
COLLEGE OF PHARMACY, TEXAS MEDICAL CENTER
UNIVERSITY OF HOUSTON
HOUSTON, TX 77204

ARCOLEO, KIMBERLY JOAN, PHD
ASSOCIATE PROFESSOR
ASSOCIATE DEAN FOR RESEARCH
SCHOOL OF NURSING
UNIVERSITY OF ROCHESTER
ROCHESTER, NY 14642

BOVBJERG, DANA H, PHD
PROFESSOR
LEADER, BIOBEHAVIORAL ONCOLOGY PROGRAM
DEPARTMENT OF PSYCHOLOGY
CANCER INSTITUTE
UNIVERSITY OF PITTSBURGH
PITTSBURGH, PA 15232

BUXTON, ORFEU M, PHD
ASSOCIATE PROFESSOR
BIOBEHAVIORAL HEALTH
PENNSYLVANIA STATE UNIVERSITY
UNIVERSITY PARK , PA 16802

CHESKIN, LAWRENCE J, MD
ASSOCIATE PROFESSOR
DEPARTMENT OF HEALTH, BEHAVIOR AND SOCIETY
JOHNS HOPKINS UNIVERSITY
BLOOMBERG SCHOOL OF PUBLIC HEALTH
BALTIMORE, MD 21205

DOZIER, ANN MARIE, RN, PHD
PROFESSOR AND CHAIR
DEPARTMENT OF PUBLIC HEALTH SCIENCES
UNIVERSITY OF ROCHESTER
ROCHESTER, NY 14642

DRENKARD, CRISTINA MARTA, MD, PHD
ASSISTANT PROFESSOR
DIVISION OF RHEUMATOLOGY
DEPARTMENT OF MEDICINE
EMORY UNIVERSITY
ATLANTA, GA 30322

FENTON, SUSAN HRACHOVY, PHD
ASSOCIATE DEAN FOR ACADEMIC AFFAIRS
SCHOOL OF BIOMEDICAL INFORMATICS
UNIVERSITY OF TEXAS
HOUSTON, TX 77030

FISCHER, STACY M, MD
ASSOCIATE PROFESSOR
UNIVERSITY OF COLORADO
HEALTH SCIENCES CENTER
DEPARTMENT OF MED/DIV OF HCPR
AURORA, CO 80011

GEORGE, MAUREEN, RN, PHD
ASSOCIATE PROFESSOR
SCHOOL OF NURSING
COLUMBIA UNIVERSITY
NEW YORK CITY, NY 10032

GIBSON, ROBERT WILLIAM, PHD
PROFESSOR AND DIRECTOR OF RESEARCH
DEPARTMENT OF EMERGENCY MEDICINE
MEDICAL COLLEGE OF GEORGIA
AUGUSTA UNIVERSITY
AUGUSTA, GA 30912

GOSS, CHRISTOPHER HOOPER, MD
PROFESSOR
DEPARTMENT OF MEDICINE
DEPARTMENT OF PEDIATRICS
UNIVERSITY OF WASHINGTON MEDICAL CENTER
SEATTLE, WA 98195

HODGSON, NANCY A, RN, PHD
ASSOCIATE PROFESSOR
DEPARTMENT OF BIOBEHAVIORAL HEALTH SCIENCES
UNIVERSITY OF PENNSYLVANIA SCHOOL OF NURSING
PHILADELPHIA, PA 19104

HUDSON, TERESA JO, PHD
DIRECTOR
DIVISION OF HEALTH SERVICES RESEARCH
PSYCHIATRIC RESEARCH INSTITUTE
UNIVERSITY OF ARKANSAS FOR MEDICAL SCIENCES
LITTLE ROCK, AK 72205

JULION, WRENETHA A, FAAN, RN, PHD
PROFESSOR
DEPARTMENT OF WOMEN, CHILDREN
AND FAMILY NURSING
COLLEGE OF NURSING
RUSH UNIVERSITY
CHICAGO, IL 60612

KALANTAR-ZADEH, KAMYAR, MD, PHD
PROFESSOR AND CHIEF
DIVISION OF NEPHROLOGY AND HYPERTENSION
DEPARTMENT OF MEDICINE
UNIVERSITY OF CALIFORNIA, IRVINE
ORANGE, CA 92868

KIM, MIYONG T, FAAN, PHD
PROFESSOR AND ASSOCIATE VICE PRESIDENT FOR
COMMUNITY HEALTH ENGAGEMENT
THE UNIVERSITY OF TEXAS AT AUSTIN
AUSTIN, TX 78701

LAWLOR, MARY C, SCD
PROFESSOR
DIVISION OF OCCUPATIONAL SCIENCE
OCCUPATIONAL THERAPY
UNIVERSITY OF SOUTHERN CALIFORNIA
LOS ANGELES, CA 90033

LEMASTER, JOSEPH W, MD
ASSOCIATE PROFESSOR
DEPARTMENT OF FAMILY MEDICINE
SCHOOL OF MEDICINE
UNIVERSITY OF KANSAS
KANSAS CITY, KS 66160

LOERZEL, VICTORIA, PHD
ASSOCIATE PROFESSOR
BEAT M AND JILL L KAHLI ENDOWED PROFESSOR
COLLEGE OF NURSING
UNIVERSITY OF CENTRAL FLORIDA
ORLANDO, FL 32826

LORI, JODY RAE, PHD
ASSOCIATE PROFESSOR
SCHOOL OF NURSING
UNIVERSITY OF MICHIGAN
ANN ARBOR, MI 48109-0482

MAGWOOD, GAYENELL SMITH, PHD
PROFESSOR
DEPARTMENT OF NURSING
COLLEGE OF NURSING
MEDICAL UNIVERSITY OF SOUTH CAROLINA
CHARLESTON, SC 29425

NESS, KIRSTEN KIMBERLIE, PHD
FULL MEMBER
DEPARTMENT OF EPIDEMIOLOGY AND CANCER CONTROL
SAINT JUDE CHILDREN'S RESEARCH HOSPITAL
MEMPHIS, TN 38105

NICHOLSON, WANDA K, MD
DIRECTOR AND PROFESSOR
PROFESSOR OF OBSTETRICS AND GYNECOLOGY
CENTER FOR WOMEN'S HEALTH RESEARCH
SCHOOL OF MEDICINE
UNIVERSITY OF NORTH CAROLINA
CHAPEL HILL, NC 27514

PORTER, STEPHEN C, MD
PROFESSOR
DEPARTMENT OF PEDIATRICS
EMERGENCY MEDICINE DIVISION
CINCINNATI CHILDREN'S HOSPITAL MEDICAL CENTER
CINCINNATI, OH 45229

QUINONES, ANA ROMAN, PHD
ASSOCIATE PROFESSOR
DEPARTMENT OF FAMILY MEDICINE
SCHOOL OF MEDICINE
OREGON HEALTH AND SCIENCES UNIVERSITY
PORTLAND, OR 97239

ROBBINS, LORRAINE BRENDA, PHD
ASSOCIATE PROFESSOR
COLLEGE OF NURSING
MICHIGAN STATE UNIVERSITY
EAST LANSING, MI 48824

SECOR, ERIC RICHARD JR, PHD
ASSOCIATE DIRECTOR
DEPARTMENT OF MEDICINE
DIVISION OF INTEGRATIVE MEDICINE
HARTFORD HOSPITAL
UNIVERSITY OF CONNECTICUT SCHOOL OF MEDICINE
HARTFORD, CT 06102

SHEPPARD, VANESSA B, PHD
PROFESSOR
HEALTH DISPARITIES RESEARCH
MASSEY CANCER CENTER
VIRGINIA COMMONWEALTH UNIVERSITY
RICHMOND, VA 23298

SIMPSON, ANN-CATHERIN NORDBO, PHD
ASSOCIATE PROFESSOR
DEPARTMENT OF HEALTHCARE LEADERSHIP
AND MANAGEMENT
COLLEGE OF HEALTH PROFESSIONS
MEDICAL UNIVERSITY OF SOUTH CAROLINA
CHARLESTON, SC 29425

STAMP, KELLY D, PHD
CHAIR AND ASSOCIATE PROFESSOR
FAMILY AND COMMUNITY NURSING
ELOISE R LEWIS EXCELLENCE PROFESSOR
SCHOOL OF NURSING
UNIVERSITY OF NORTH CAROLINA GREENSBORO
GREENSBORO, NC 27402

SZALACHA, LAURA A, EDD
PROFESSOR
COLLEGE OF NURSING
UNIVERSITY OF ARIZONA
TUCSON, AZ 85721

TANCREDI, DANIEL JOSEPH, PHD
ASSOCIATE PROFESSOR
DEPARTMENT OF PEDIATRICS
CENTER FOR HEALTHCARE POLICY AND RESEARCH
UNIVERSITY OF CALIFORNIA, DAVIS
SACRAMENTO, CA 95817

UPHOLD, CONSTANCE R, PHD
ASSOCIATE DIRECTOR, GRECC
IMPLEMENTATION AND OUTCOMES RES
GERIATRIC RESEARCH EDUCATION CLINICAL CENTER
GAINESVILLE VA MEDICAL CENTER
UNIVERSITY OF FLORIDA COLLEGE OF MEDICINE
GAINESVILLE, FL 32608

WANG, DONGWEN, PHD
PROFESSOR
DEPARTMENT OF BIOMEDICAL INFORMATICS
ARIZONA STATE UNIVERSITY
SCOTTSDALE, AZ 85259

WETHINGTON, ELAINE, PHD
PROFESSOR
DEPARTMENT OF HUMAN
DEVELOPMENT AND SOCIOLOGY
CORNELL UNIVERSITY MVR HALL
ITHACA, NY 14853

YEN, PO-YIN, PHD
ASSISTANT PROFESSOR
DEPARTMENT OF MEDICINE; DIVISION OF GENERAL
MEDICAL SCIENCES; INSTITUTE FOR INFORMATICS
GOLDFARB SCHOOL OF NURSING, BJC HEALTHCARE
WASHINGTON UNIVERSITY IN ST LOUIS
ST LOUIS, MO 63110

SCIENTIFIC REVIEW OFFICER

HARE, MARTHA L, RN, PHD
SCIENTIFIC REVIEW OFFICER
CENTER FOR SCIENTIFIC REVIEW
NATIONAL INSTITUTES OF HEALTH
BETHESDA, MD 20892

EXTRAMURAL SUPPORT ASSISTANT

JONES, BELINDA
EXTRAMURAL SUPPORT ASSISTANT
CENTER FOR SCIENTIFIC REVIEW
NATIONAL INSTITUTES OF HEALTH
BETHESDA, MD 20892

Consultants are required to absent themselves from the room during the review of any application if their presence would constitute or appear to constitute a conflict of interest.
